# Morphological Analysis of Size and Shape (MASS): An integrative software program for morphometric analyses of leaves

**DOI:** 10.1002/aps3.11288

**Published:** 2019-09-19

**Authors:** Tya S. Chuanromanee, James I. Cohen, Gillian L. Ryan

**Affiliations:** ^1^ Department of Computer Science Kettering University 1700 University Avenue Flint Michigan 48532 USA; ^2^ Present address: Department of Computer Science and Engineering University of Notre Dame 384 Fitzpatrick Hall Notre Dame Indiana 46556 USA; ^3^ Department of Applied Biology Kettering University 1700 University Avenue Flint Michigan 48532 USA; ^4^ Department of Physics Kettering University 1700 University Avenue Flint Michigan 48532 USA

**Keywords:** landmark analysis, leaves, morphometrics, outline analysis

## Abstract

**Premise:**

Morphometric analysis is a common approach for comparing and categorizing botanical samples; however, completing a suite of analyses using existing tools may require a multi‐stage, multi‐program process. To facilitate streamlined analysis within a single program, Morphological Analysis of Size and Shape (MASS) for leaves was developed. Its utility is demonstrated using exemplar leaf samples from *Acer saccharum*,* Malus domestica*, and *Lithospermum*.

**Methods:**

Exemplar samples were obtained from across a single tree (*Acer saccharum*), three trees in the same species (*Malus domestica*), and online, digitized herbarium specimens (*Lithospermum*). MASS was used to complete simple geometric measurements of samples, such as length and area, as well as geometric morphological analyses including elliptical Fourier and Procrustes analyses. Principal component analysis (PCA) of data was also completed within the same program.

**Results:**

MASS is capable of making desired measurements and analyzing traditional morphometric data as well as landmark and outline data.

**Discussion:**

Using MASS, differences were observed among leaves of the three studied taxa, but only in *Malus domestica* were differences statistically significant or correlated with other morphological features. In the future, MASS could be applied for analysis of other two‐dimensional organs and structures. MASS is available for download at https://github.com/gillianlynnryan/MASS.

Morphometric analysis is a useful methodology employed in multiple areas of plant biology, ranging from ecology (Gómez et al., 2016) and evolution (Rose et al., 2016) to genetics (Leiboff et al., 2015) and agriculture (de Oliveira et al., 2016). Currently, two approaches dominate morphometric studies—traditional morphometrics and geometric morphometrics. Traditional morphometrics involves measuring multiple quantitative characters among a set of individuals (usually lengths, widths, and ratios) and using inferential statistics (e.g., ANOVA or multivariate statistical analyses) on the collected data to identify cohesive groups of individuals (Andersson, 1994; Marhold, 2011). Andersson (1994), Boyd (2002), Bateman and Rudall (2006), Marhold (2011), Garcia‐Cruz and Sosa (2006), Jimenez‐Mejias et al. (2017), and many others have used this approach to delineate species and assess morphological variation across the geographic range of species. Geometric morphometrics, on the other hand, involves the use of quantitative representation of shape, with landmarks and/or outlines, to compare morphological features, such as leaves, flowers, and seeds, and it uses images of these features as a starting point for analysis. Geometric morphometrics employs landmarks (homologous features of an organ) or semi‐landmarks for Procrustes analysis and outlines of shapes for elliptical Fourier analysis (Kuhl and Giardina, 1982). In doing so, geometric morphometrics takes advantage of a larger number of characteristics than may be available (and easily discernable) in traditional morphometric approaches and also allows for variation in organ size and shape, which may be challenging to include in analyses that use only traditional morphometrics. Geometric morphometrics has increased in popularity during the past decade in multiple fields (Adams et al., 2013; Manacorda and Asurmendi, 2018), and, as with traditional morphometrics, has allowed for an understanding of patterns of species diversity (Chitwood and Otoni, 2017; Klein et al., 2017). Indeed, given the number of digitized herbarium specimens currently publicly available (e.g., JSTOR's Global Plants database [https://plants.jstor.org] and the SEINet specimen database [http://swbiodiversity.org/seinet/collections/index.php]), it is possible to take advantage of scanned specimens for geometric morphometrics.

One challenge with the implementation of geometric morphometric analyses is that a single study can involve the use of multiple software packages on different platforms, and these multi‐program workflows can result in a steep learning curve for newcomers as well as introduce error due to the use of multiple file types that require manual pre‐ and post‐processing at various analysis stages. This use of multiple programs was exemplified in a recent paper describing a protocol to examine the geometric morphometrics of flower symmetry. Savriama (2018) outlined multiple software packages for preparing images for landmark analysis, such as tpsUtil and tpsDig2 (Rohlf, 2015), followed by MorphoJ (Klingenberg, 2011) and R (R Core Team, 2013) to analyze the landmark data. This resulted in four software programs necessary to conduct an analysis of landmarks, and additional software, such as geomorph (Adams et al., 2016), SHAPE (Iwata and Ukai, 2002), momocs (Bonhomme et al., 2014), and DiaOutline (Wishkerman and Hamilton, 2018), may be required to undertake analysis of the outline of morphological features (e.g., ImageJ to convert a color image to a binary image, SHAPE for chain‐code generation, and momocs for analysis of the shape outline). Consequently, while geometric morphometrics can provide important information on morphological variation and diversity, users frequently must master not only the methods themselves but also multiple software programs for a complete analysis. This can increase the amount of time for training for research personnel, and manual collection and manipulation of data may introduce additional sources of error. The present study provides a software program, Morphological Analysis of Size and Shape **(**MASS) that integrates all steps of a basic geometric morphometric analysis. This allows for an all‐in‐one platform to generate results from images of organs. To date, MASS has been used to examine leaf shape from three different groups of plants, and the examples are described in the present study, with different types of analyses employed depending on the leaf shape.

## METHODS

Leaves from one tree of *Acer saccharum* Marshall (Sapindaceae) were used to investigate variation within an individual as well as to compare the results of the Procrustes analysis to those of elliptical Fourier analysis. With leaves from three trees of *Malus domestica* (Suckow) Borkh. (Rosaceae), variation within and among individuals was examined using elliptical Fourier analysis; Procrustes analysis was not possible given a lack of identifiable homologous landmarks. Within‐genus leaf variation was examined in *Lithospermum* L. (Boraginaceae), a genus that includes qualitative diversity in leaves and flowers (Cohen, 2018), but in which leaf outlines have not been utilized to further understand vegetative diversity among the species. In *Lithospermum*, some species produce leaves with only a midvein (i.e., one primary vein) and no noticeable secondary veins, whereas other species develop leaves with a midvein and noticeable secondary veins (Cohen, 2016). Additionally, particular patterns of leaf venation are often associated with specific types of flowers, such as leaves with evident secondary venation being produced by species with long corollas with exserted anthers and stigmas (i.e., *Macromeria*‐type flowers of Cohen, 2016). Shape variation among leaves with different types of venation or associated with particular flower types has yet to be examined using geometric morphometric methodologies, and undertaking this type of study will add to the understanding of the evolution and variation of floral morphospace of the genus *Lithospermum* (Cohen, 2016).

### Software

**Figure 1:**
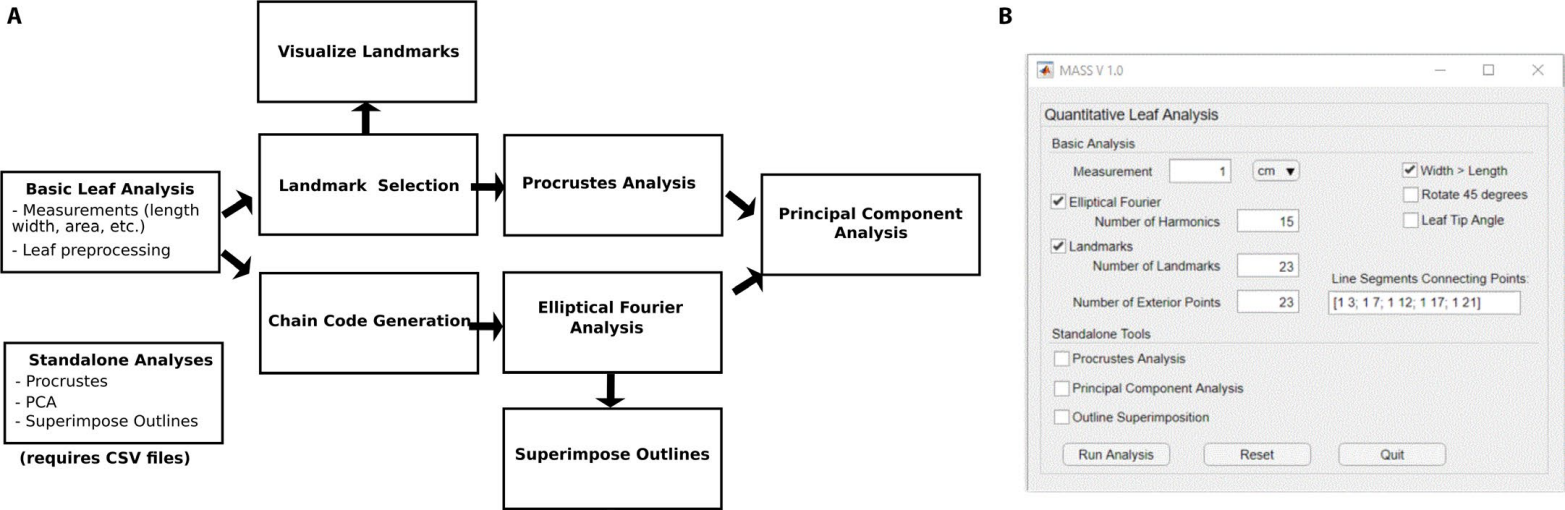
Data collection and analysis stream for MASS. (A) The workflow for MASS data collection and analysis includes landmark and chain code generation, which can be used to compute Procrustes and elliptical Fourier analyses, respectively. Data from both of these analysis pipelines can be further processed using principal component analysis (PCA). (B) The graphical user interface (GUI) for the MASS tool allows users to select single or multiple analysis streams, as well as specify the number of landmarks and harmonics to include in the analyses.

The MASS tool was developed in MATLAB (MATLAB, 2018) but has been compiled to be run as a standalone application. As a result, this tool may be used even by users who do not have MATLAB licenses. The app has a user‐friendly graphical user interface (GUI) that allows point‐and‐click processing of images, but as it was developed in MATLAB, it is able to leverage the many libraries and functions already available in the MATLAB environment. The program is modular, so that the user may select which analyses to be completed a la carte, and MASS can also support stand‐alone post‐analysis of files generated using the tool without restarting the entire analysis sequence, as shown in Fig. 1A. MASS has been designed to be semi‐automated, so that user inputs are required, but some steps are fully automated based on those inputs. This blend of automation and manual entry allows for manual troubleshooting or intervention at some analysis stages, while minimizing processing error in others.

The analysis sequence begins with the user's selection of an image file to be analyzed. The image employed needs to be of sufficiently high resolution in order to be useful for the research question the user would like to ask (i.e., small and/or narrow objects can be used with MASS as long as the image has sufficient resolution to clearly delineate the object's features of interest). Upon image selection, the user is prompted to select desired analyses within the GUI, as well as specify the size‐scale for image calibration. An image of the GUI is shown in Fig. 1B, with both elliptical Fourier and landmark analyses selected. Upon initiating the analysis, the user will be prompted to select a length scale (i.e., on a ruler) within the image using their mouse, which the MASS tool uses to establish the physical size scale of each pixel in the image. The MASS tool allows users to process multiple samples per image, and upon the completion of each sample will inquire if the user would like to analyze another so that this scaling process need only be completed once per image file. Because MASS was developed using leaves as exemplar specimens, the methods here will describe the analysis of leaves, but the tool can be used to study other organs, such as flowers or seeds, with similar methodology.

**Figure 2:**
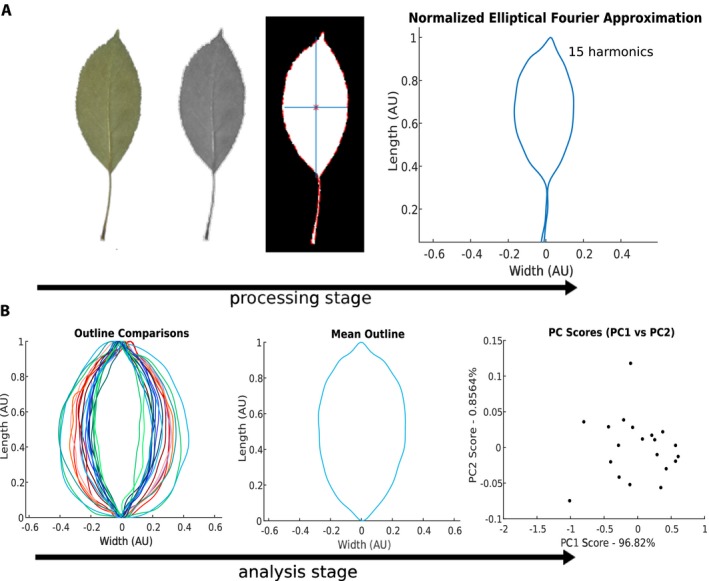
Typical leaf shape processing and analysis stages utilized by MASS with *Malus domestica* leaves as an example. (A) Stages of image processing for *M. domestica* leaf, displayed from the raw image of a leaf (far left) to a normalized contour characterized by elliptical Fourier descriptors (EFD, far right). During processing, MASS converts the original image to grayscale, followed by a thresholded black‐and‐white image. Superimposed on the black‐and‐white image are the leaf's centroid, indicated by an asterisk, the leaf's edge, indicated by a red dotted line, and also the primary axes of the leaf, indicated in blue. (B) Stages of comparative analysis of leaf shape showing multiple leaf outlines generated using EFD (left), the generation of a mean leaf outline (center), and principal component analysis (PCA) of the EFD coefficients (right).

Upon loading and scaling the image, the user will be prompted to select a leaf for analysis, as is shown in Fig. 2A. Images may contain multiple leaves, and this step is not automated in order to allow for flexibility. This selection is accomplished by using the mouse to indicate points on a closed contour, creating a bounded region that encloses the leaf of interest. The image is then converted to grayscale. If selected, the user may also make measurements of the angle of the leaf tip at this time, by selecting three points to define the angle of the desired leaf feature when prompted. The MASS tool uses thresholding to then binarize the selected portion of the image, creating a new black‐and‐white image representing the exterior and interior of the leaf, respectively, bounded by a dotted red line indicating the leaf margin. Although the petiole is included in the processing steps displayed in Fig. 2A, selection should exclude features not to be included for comparative analysis (e.g., the petiole if only leaf shape is of interest), as well as fragments of other leaves or plant matter, as they will distort analysis results. From the black‐and‐white image, the MASS tool calculates the centroid of the leaf as well as its principal axes, and rotates the leaf so it is aligned with its long axis along the vertical direction. In Fig. 2A, the primary axes are indicated by blue lines in the black‐and‐white thresholded image. In the case that the leaves of a species are typically wider than they are tall, the user may opt to rotate the leaves so that the long axis is along the horizontal direction by indicating that ‘Width > Length’ in the GUI before analysis. From this standard orientation, more sophisticated geometric analyses may be applied to each leaf.

After binarization and rotation, this new binary image is used to provide basic measurements of leaf shape features. Measurements of leaf height and width are provided by fitting the now‐upright leaf with a bounding box, whose height and width correspond to the height and width of the chosen leaf. The leaf area is also provided, calculated from the number of white pixels within the bounding box. The fluctuating asymmetry (FA) of each leaf is also calculated, providing a quantitative measure of the left–right asymmetry of each leaf (e.g., Rozefelds and Pace, 2018). The FA is calculated as

$$FA=2\left(\frac{WL-WR}{WL+WR}\right),$$

where *WL* and *WR* indicate the distance from the central vertical axis of the leaf to the left and right edges of the leaf, respectively. The quantity is normalized by the total width of leaf, and a value of FA = 0 indicates a perfectly symmetric region on the leaf, whereas a negative value indicates it skews to the right of center and a positive value indicates it skews to the left of center. MASS reports the average absolute FA value for each leaf, providing an average (positive) measure of symmetry for each leaf.

In addition to measurements of length, width, area, and asymmetry, the binarized leaf image is also the starting point for the calculation of elliptical Fourier descriptors (EFDs) for each leaf. Elliptical Fourier analysis is a common approach for comparing and contrasting objects of similar, but different geometries, as the Fourier coefficients generated in this type of an analysis are invariant with translation, rotation, and dilation of a closed contour representing an object's shape (Kuhl and Giardina, 1982; Chitwood and Otoni, 2017; Klein et al., 2017). During elliptical Fourier analysis, leaves are scaled to a normalized height, and then nearest‐neighbor searching throughout the binarized image identifies pixels at the boundary between the inside and outside of the leaf, which comprise the closed contour on which this analysis is based. The closed contour depicting the boundary of the leaf is converted into a chain code, as described in Kuhl and Giardina (1982), which is then analyzed using the Elliptical Fourier for Shape Analysis tool available in MATLAB's File Exchange (Manurung, 2016).

**Figure 3:**
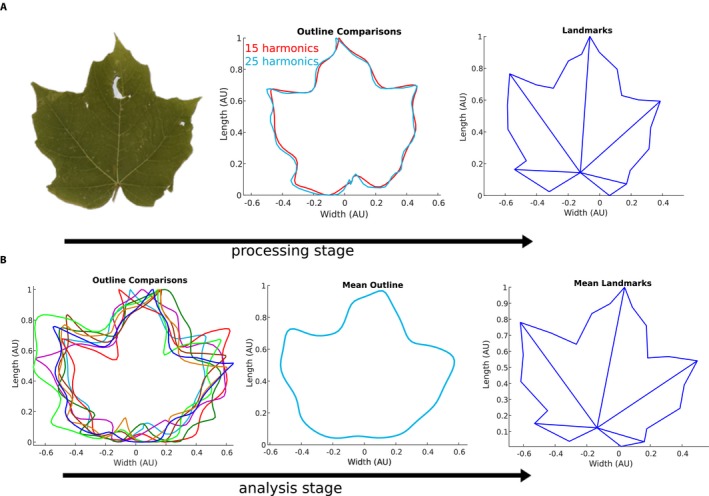
Typical landmark processing and analysis stages utilized by MASS for *Acer saccharum* leaves. (A) Stages of image processing for *A. saccharum* leaf, displayed from the raw image of a leaf (left) to a normalized contour characterized by elliptical Fourier descriptors (EFD) to landmark representation (right). Two EFD outlines are provided in the middle panel, displaying results using *N* = 15 and *N* = 25 harmonics. (B) Stages of comparative analysis of leaf shape showing sample multiple leaf outlines generated using EFD (left), the generation of a mean leaf outline (center), and a representative average landmark set generated by Procrustes analysis of the population landmarks over 171 leaves (right).

The MATLAB EFD tool fits a Fourier series to the contour described by the chain code, including a user‐defined number of harmonics in the series, *N*, as shown in the GUI (Fig. 1B). As indicated in Kuhl and Giardina (1982), an increased number of harmonics improves the fit of the series to the contour, but requires additional computation for smaller and smaller gains in overall accuracy with each increase in the number of harmonics. This is demonstrated in Fig. 3A, which compares the EFD‐generated outline for a maple leaf using *N* = 15 or 25 harmonics. We find that *N* = 15–20 harmonics are typically sufficient to fit the leaves examined in the present study, although leaves with smaller, finer features may require additional harmonics to capture these details.

The MASS tool displays the elliptical Fourier approximation of the edge contour upon its calculation to allow such troubleshooting. The EFD tool returns the four Fourier coefficients for each harmonic, exported to a comma‐separated values (CSV) file, which allows comparison of the coefficients across populations of leaves. After this fitting, principal component analysis (PCA) allows for the comparison of sample outlines across populations of leaves, an example of which is shown in Fig. 2B. The coordinates of the leaf outline are saved in a similar manner to allow later comparison across populations of leaves, and can be compared using the Outline Superposition option shown in Fig. 1B. Output of this analysis, including a mean leaf outline, is displayed in Figs. 2B and 3B.

Although EFD is a useful tool for comparing overall leaf shape and morphology, it does not provide a metric for comparing the internal structures of leaves or specific homologous features of leaves. For this, we use landmark selection and analysis. If selected, this analysis occurs before binarization, on the grayscale image. Landmark selection is a manual process, and the number of landmarks for a given leaf type can be specified by the user at the start of the analysis in the GUI (Fig. 1). Users should plan the order of the landmark selection to preserve the landmark number assigned to given features. During landmark visualization, MASS generates line segments connecting specified landmarks, the input for which is shown in Fig. 1B. Within the GUI, each connected landmark pair is listed within square brackets, with pairs separated by a semicolon. In this instance, MASS has been initialized to analyze leaves of *A. saccharum*, in which 23 landmarks were identified, and line segments connect landmarks 1 and 3, 1 and 7, 1 and 12, and so on. The MASS tool exports the coordinates of the landmarks, as well as an image displaying the connected landmarks. An example of MASS landmark identification for a leaf of *A. saccharum* is shown in Fig. 3A. After the landmark identification, users may opt to complete a Procrustes analysis on their data, which compares the distribution of shapes across multiple samples and generates a standard set of landmarks across samples. An example of the mean landmarks used in this analysis is shown in Fig. 3B. Upon completion of the Procrustes analysis, users may also perform PCA on this data. The MASS software, instructions for the software, and sample data are available for download at https://github.com/gillianlynnryan/MASS.

### Plant material and morphometric analyses

To demonstrate the efficacy of the MASS tool, 172 and 992 leaves were analyzed from recent collections of one tree of *A. saccharum* from Mott Park in Flint, Michigan, USA, and three trees of *M. domestica* at For‐Mar Nature Preserve and Arboretum in Burton, Michigan, USA, respectively. Leaves were collected, pressed, and imaged, and voucher specimens (Cohen 485, 486) were deposited in the University of Michigan Herbarium (MICH). To demonstrate the flexibility of the MASS tool, a total of 88 leaves from digitized herbarium specimens from online repositories were collected across 26 species, 25 of *Lithospermum* and one of the related genus *Buglossoides* Moench (Boraginaceae), and subsequently analyzed.

Basic descriptive statistics (i.e., length, width, and fluctuating asymmetry) were collected for all leaves, and the results were graphed. The distributions were compared using both one‐ and two‐tailed *t*‐tests, with similar *P* values generated for both tests. EFD was conducted for all leaves with *N* = 15 harmonics for each leaf, and given that landmarks could be identified for the leaves of *A. saccharum*, Procrustes analysis was also undertaken for this species. The 23 landmarks were identified as the points of the lobes of the leaves (Fig. 3). The results from EFD and Procrustes analyses were examined with PCA and graphed to visualize the variation in leaf morphology. For analyses of *M. domestica*, the leaves were analyzed per tree, and for *Lithospermum*, the leaves were analyzed based on the type of leaf venation and by the flower type (after Cohen, 2016). PCA was conducted on the results of the EFD and Procrustes analysis.

## RESULTS

**Figure 4:**
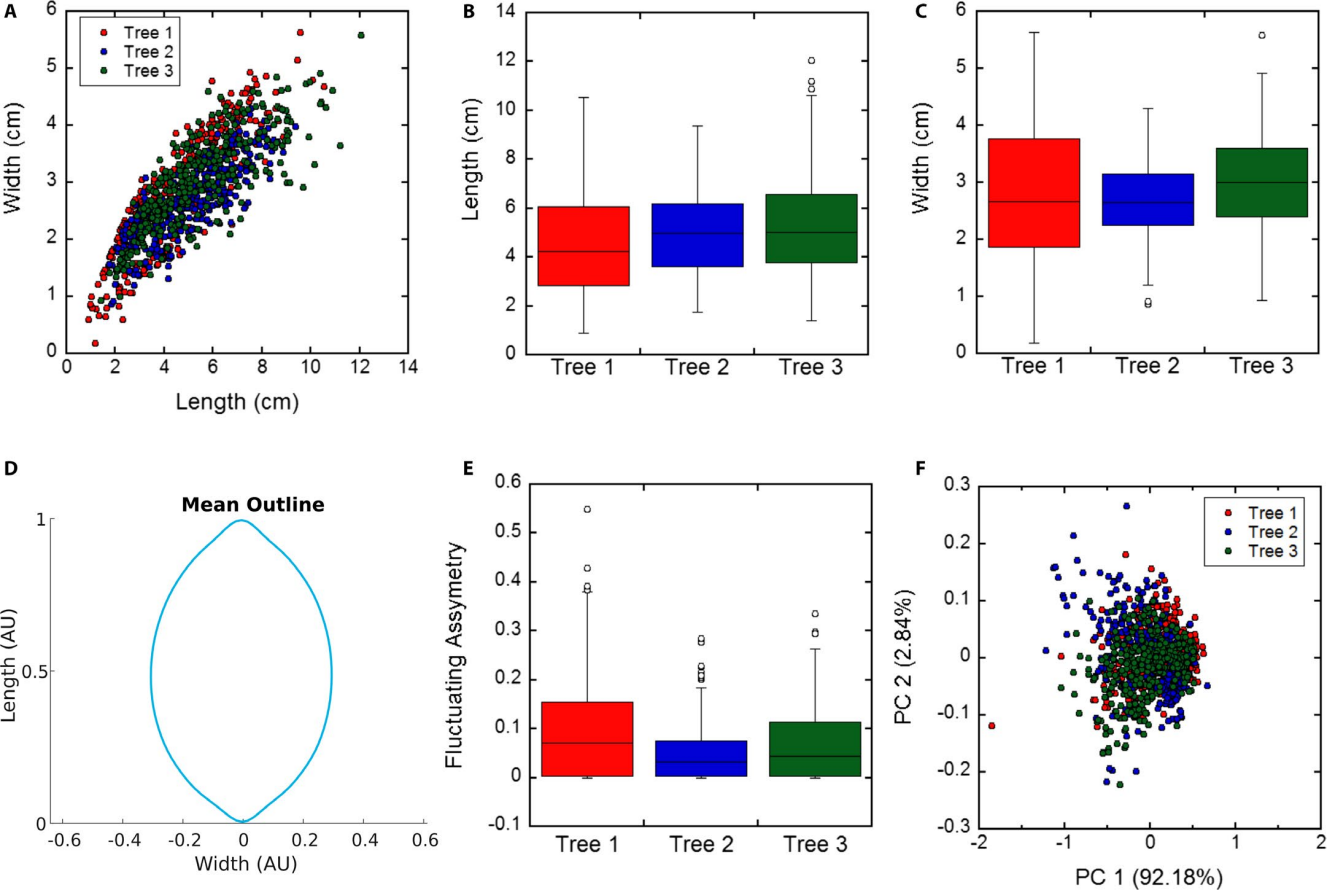
Results from analysis of leaves from three *Malus domestica* trees. (A) A strong correlation is noted between the length and width of the sampled *M. domestica* leaves. (B) Leaf length distributions. Average leaf lengths are 4.5, 5.0, and 5.3 cm, respectively, and *P* < 0.05 for all three pairs of trees. (C) Leaf width distributions. Average leaf widths are 2.8, 2.7, and 3.0 cm, respectively, and *P* < 0.10 for all three pairs of trees. (D) The mean normalized leaf outline averaged over 992 samples from all three trees. (E) Fluctuating asymmetry (FA) distributions. Average absolute FA are 0.094, 0.051, and 0.069, respectively, and *P* < 0.05 for all three pairs of trees. (F) Principal component analysis (PCA) of the elliptical Fourier descriptors generated using *N* = 15 harmonics. A total of 269, 365, and 359 leaves were sampled, respectively, for the length, width, and FA measurements.

Results shown in Fig. 4A indicate a strong correlation between the length and width of the *M. domestica* leaves across all three trees, but the length distributions (Fig. 4B) were found to be statistically different between trees. Similarly, the width distributions (Fig. 4C) of trees 1 and 3 and trees 2 and 3 were found to be statistically different, but *P* > 0.05 for the width distributions of trees 1 and 2, suggesting that these width distributions were not as dissimilar. The distribution of mean (absolute) fluctuating asymmetry (FA), which provides a measure of the asymmetry of each leaf, is shown in Fig. 4E. On average, the leaves displayed a small amount of asymmetry, and the FA was statistically significantly different between trees. Although all of these basic descriptive statistics are statistically significant differences between the pairs of trees, the amount of variation is minimal.

While simple geometric analyses were possible directly from leaf images, EFD allowed for more sophisticated analyses to be conducted. As each harmonic requires four coefficients to describe it, each individual outline with *N* = 15 harmonics requires a total of 60 fit parameters. The average leaf outline, across all three trees, is shown in Fig. 4D. PCA of the EFD coefficients for all of the *M. domestica* leaves is shown in Fig. 4F, and most of the variation is observed in the first principal component (ca. 92%). No overt separation between the leaves of the three trees is noted.

**Figure 5:**
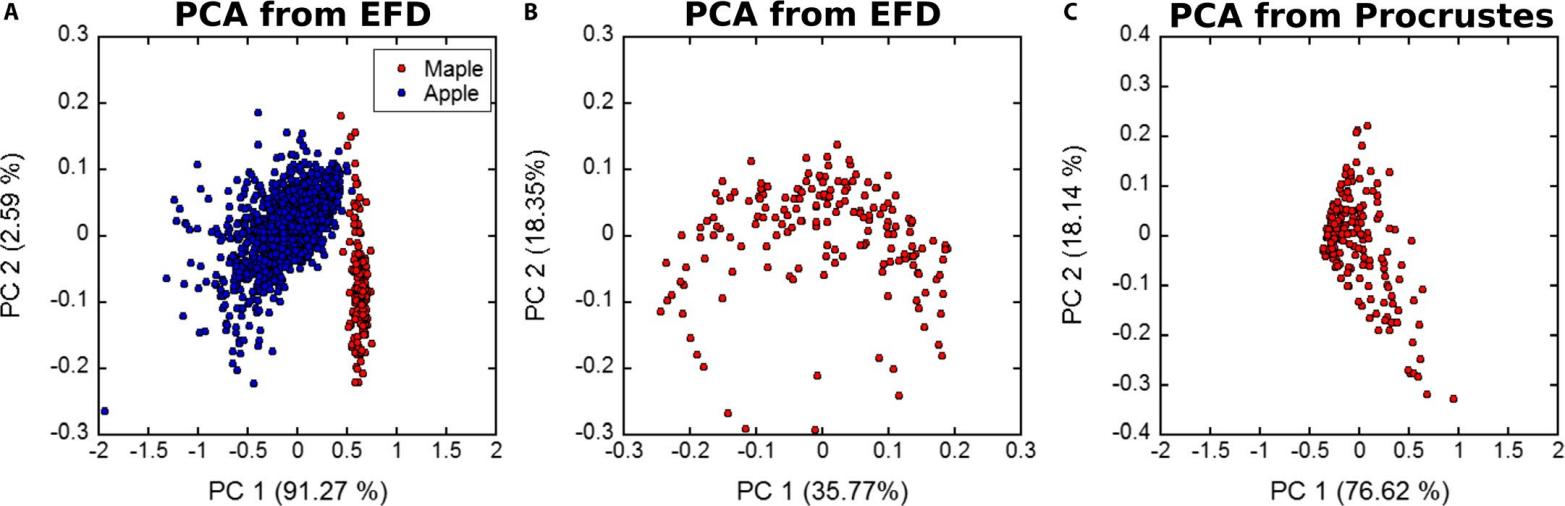
Principal component analysis (PCA) of leaves. (A) PCA of elliptical Fourier descriptors for 171 * Acer saccharum* (red) and 992 *Malus domestica* (blue) leaves. (B) PCA of elliptical Fourier descriptors for 171 *A. saccharum* leaves. (C) PCA of the landmark shift distances for all landmarks across 171 *A. saccharum* leaf samples based on the representative landmarks shown in Fig. 3B.

For *A. saccharum*, both outline and landmark analyses were conducted. Variation in landmark positions was characterized using a Procrustes analysis, and the shifts between the landmarks and the representative set were collected. The PCA of the results of the Procrustes analysis demonstrates that most of the variation is observed in the first and second principal components, ca. 77% and 18%, respectively. There is greater variation in the morphospace with the use of landmarks and Procrustes analysis compared to outlines and EFD (Fig. 5B, C). With the results of the EFD, PCA was conducted to compare the *Acer* and *Malus* leaves. The results are shown in Fig. 5A, where a distinct separation of the two populations is apparent, with *Malus* leaves having greater variation compared to the *Acer* leaves.

**Figure 6:**
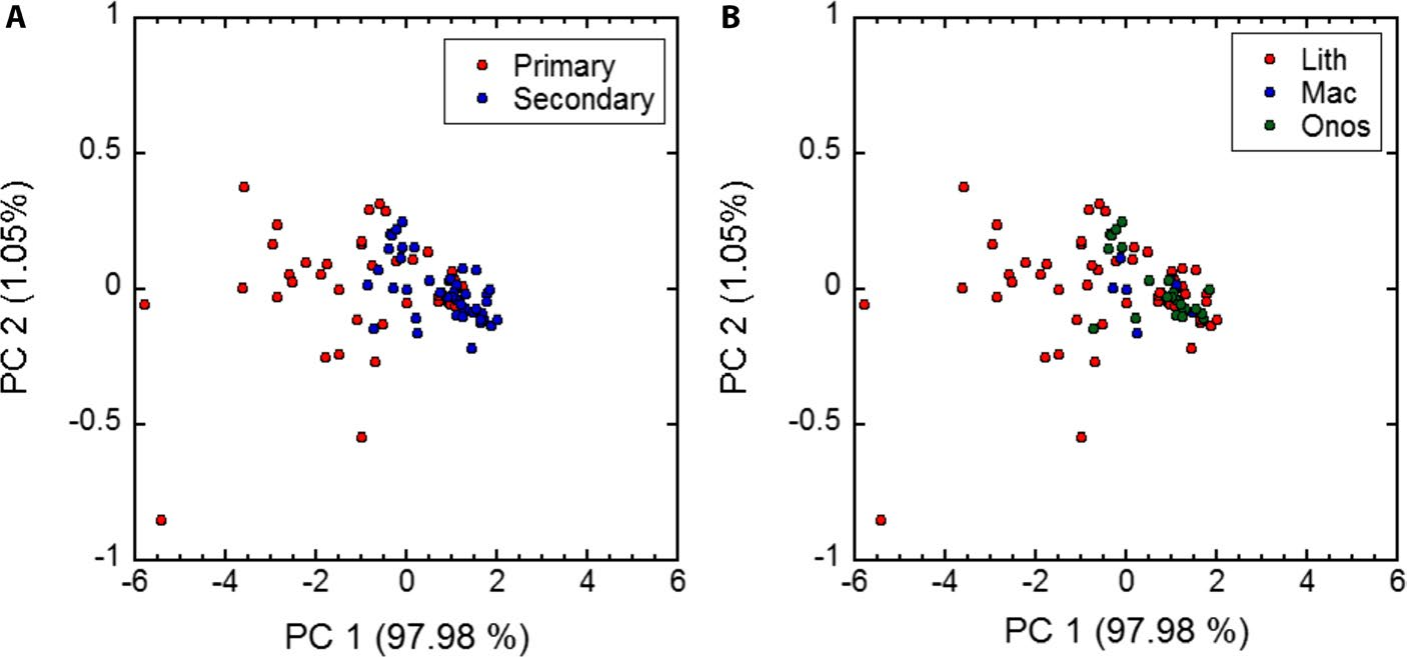
Principal component analysis (PCA) of elliptical Fourier descriptors of *Lithospermum* leaves sorted by (A) leaf venation pattern (i.e., primary or secondary venation) and (B) flower type after Cohen (2016).

In *Lithospermum* and relatives, the leaves examined with EFD and PCA show little relationship with pattern of venation or type of flower, and the first principal component includes the vast majority of the variation (ca. 98%) (Fig. 6). The leaves with only primary venation encompass greater morphospace than those with evident secondary venation, and the two groups of leaves overlap sufficiently to result in a lack of distinction between venation patterns. Similarly, leaf shape is not associated with flower type; although leaves of species with *Macromeria*‐ and *Onosmodium*‐type flowers are more similar to each other than to those for species that bear *Lithospermum*‐type flowers.

## DISCUSSION

### Validation of MASS

In *A. saccharum*, little morphological variation is observed, which is unsurprising given that all examined leaves were from the same tree. This lack of variation is especially apparent when compared to the leaves of *M. domestica* (Fig. 5A). *Acer saccharum* was the only studied species in which both landmarks and outlines were employed given that the leaves had homologous features that could be identified. From the leaves of *A. saccharum*, the landmarks provided greater variation among the samples, albeit only by a little. Chitwood and Otoni (2017) observed a similar result when examining species of *Passiflora* L., using both landmarks and outlines. Whereas the landmarks provided greater total variation among the leaves, the outline analyses did allow for separation and identification of morphological variation among the samples (Fig. 5).

In *M. domestica*, the pairs of individuals were found to be statistically significant, but the differences among the trees were fairly small, even with almost 1000 leaves sampled. It is notable that the individuals did exhibit some variation, such as tree 1 having the greatest range in length and width and tree 3 having the longest and widest leaves (Fig. 4). In general, these differences in leaf characteristics were a few millimeters compared to the overall length and width of 4–5 cm and 2–3 cm, respectively, among the leaves. Although the results from the present study did not result in any observed differences, a broader study of leaf shape in multiple cultivars of *M. domestica* identified leaf variation, with most of the variation in the first principal component in the aspect ratio (Migicovsky et al., 2017). Migicovsky et al. (2017) state that a large amount of the variation in the leaves of *M. domestica* is based on this length‐to‐width ratio, a similar result observed in the present study, with the lack of variation likely the result of using three individuals of the same putative cultivar.

In *Lithospermum*, the results of the EFD provide for no distinct clusters of species based on leaf venation patterns or flower type. In the genus, patterns of leaf venation can be readily observed, and species with larger leaves tend to have secondary venation while those with smaller leaves bear leaves with only a midvein (Cohen, 2016). This is not always the case though, and some species, such as *L. chihuahuanum* J. I. Cohen and *L. guatemalense* Donn. Sm., produce smaller leaves with secondary venation (Cohen, 2018). Therefore, patterns of leaf venation do not appear to be tightly connected with other features of leaf size and shape. Similarly, while flower morphology can help identify three groups of *Lithospermum* (Cohen, 2016), leaf types associated with these flower types (midvein with *Lithospermum*‐type flowers and a midvein and evident secondary veins with *Onosmodium*‐ and *Macromeria*‐type flowers) do not sufficiently differ in size and shape to also diagnose the species assigned to these groups.

### Utility of MASS

MASS streamlines the process of morphometric analysis, and this has been validated with studies on leaves from multiple species. While three or more different software programs may be necessary for landmark and Procrustes analysis and outline and EFD (Chitwood and Otoni, 2017; Klein et al., 2017; Savriama, 2018), with MASS, only one program is needed for a morphometric study. Indeed, the MASS program consolidates multiple data acquisition and computation steps into an analysis pipeline within a single tool, with no need for reformatting files between analysis stages. This should decrease the barrier of entry to undertake morphometric studies as the number of software programs not only needed but also required to learn is minimized. Additionally, data are saved in commonly used formats that allow users to export the data to other tools for post‐analysis and also provide for the PCA or Procrustes analysis of data generated from other programs. As with all software for image analysis, resolution can be a concern; therefore, it is necessary to ensure that images are of sufficiently high resolution to address the morphometric questions researchers are seeking to analyze. MASS is a versatile and comprehensive tool for analyzing and comparing morphological features of plant organs. To date, it has been used to examine leaf morphology of three taxa for geometric morphometrics, and we believe its utility extends to examination of other sample types; however, we have yet to explicitly validate the software on other plant structures.

MASS is quite useful for the types of basic analyses we have conducted, and the software employs standard methods of data analysis in the field, such as Procrustes analysis and PCA, allowing for comparison with results from other studies. It is important to note that MASS is a general tool for study of morphometrics, and other tools and software may provide greater utility for organs that require more sophisticated analyses, such as examination of asymmetric corollas (Savriama, 2018).

Future work will focus on two aspects of the software. First, we will try to incorporate additional analyses for morphometric data, such as multivariate regression for the study of allometry (Klingenberg, 2016). Second, we will continue investigating leaves of other species to further refine the software program and will also use flowers, fruits, and seeds for morphometric analyses to explore the use of MASS for other types of plant organs. These organs can provide additional data for morphometric studies (Bateman and Rudall, 2006), but can be challenging to orient appropriately, particularly with the use of digitized herbarium specimens. Overall, MASS is a useful software program as it integrates the multiple components of a workflow for morphometric analyses, making it easier for researchers to engage in this type of intriguing research.

## AUTHOR CONTRIBUTIONS

G.L.R. and J.I.C. conceived the project. G.L.R. managed the project. T.S.C. led software development, testing, and data acquisition. Data were confirmed and interpreted by G.L.R. and J.I.C. G.L.R. and J.I.C. wrote the manuscript, which T.S.C. edited.

## Data Availability

MASS is available for download at https://github.com/gillianlynnryan/MASS.
